# Update on the diagnosis and treatment of pericardial diseases: a position paper of the Italian Society of Cardiology in collaboration with the study group on cardiomyopathies and pericardial diseases

**DOI:** 10.2459/JCM.0000000000001684

**Published:** 2024-11-25

**Authors:** Massimo Imazio, Valentino Collini, Alberto Aimo, Camillo Autore, Barbara Bauce, Elena Biagini, Francesco Cappelli, Silvia Castelletti, Flavio D’Ascenzi, Cesare De Gregorio, Giuseppe Limongelli, Francesca Marzo, Marco Merlo, Beatrice Musumeci, Stefania Paolillo, Giacomo Tini, Roberto Pedrinelli, Pasquale Perrone Filardi, Gianfranco Sinagra

**Affiliations:** a Department of Medicine (DMED), University of Udine; b Cardiothoracic Department, University Hospital Santa Maria della Misericordia, ASUFC, Udine; c Scuola Superiore Sant’Anna, Fondazione Monasterio, Pisa; d Department of Clinical and Molecular Medicine, Sapienza University, Rome; e Department of Cardiac, Thoracic and Vascular Sciences and Public Health, University of Padua, Padua; f Cardiology Unit, IRCSS Azienda Ospedaliero-Universitaria di Bologna, Bologna; g Tuscan Regional Amyloidosis Centre, Careggi University Hospital, Florence; h Cardiology Department, Istituto Auxologico Italiano IRCSS, Milan; i Department of Medical Biotechnologies, Division of Cardiology, University of Siena, Siena; j Department of Clinical and Experimental Medicine, University Hospital of Messina, Messina; k Inherited and Rare Cardiovascular Diseases, Department of Translational Medical Sciences, University of Campania Luigi Vanvitelli, Monaldi Hospital, Naples; l Cardiology Unit, Infermi Hospital, Rimini; m Cardiovascular Department, ‘Azienda Sanitaria Universitaria Giuliano-Isontina’, and University of Trieste, Trieste; n Department of Advanced Biomedical Sciences, Italian Society of Cardiology, Federico II University of Naples, Naples; o Cardiac, Thoracic and Vascular Department, University of Pisa, Pisa, Italy

**Keywords:** constrictive pericarditis, diagnosis, multimodality imaging, pericardial effusion, pericarditis, therapy

## Abstract

The knowledge of pericardial diseases has now improved, including prospective and retrospective cohort studies focusing on the pathogenesis, diagnosis, treatment, and outcomes. The complex interplay between genetic predisposition (especially for autoinflammatory conditions), inflammation, and autoimmunity is now known to trigger recurrences of pericarditis. Moreover, diagnostic capabilities have improved with the implementation of multimodality imaging, particularly cardiac magnetic resonance (CMR), to detect and monitor pericardial inflammation, to allow diagnosis in more complicated cases, and tailor the duration of therapy based on objective parameters. A new class of drugs, the anti-IL-1 agents, have been introduced for patients with an inflammatory phenotype of presentation, and not responding to conventional anti-inflammatory therapies, including NSAID, colchicine, and corticosteroids. At present, the clinical management of pericardial diseases is definitely on the road of evidence-based medicine with new ongoing European guidelines focusing on the spectrum of inflammatory myocardial and pericardial syndromes.

## Graphical abstract

**Figure:**
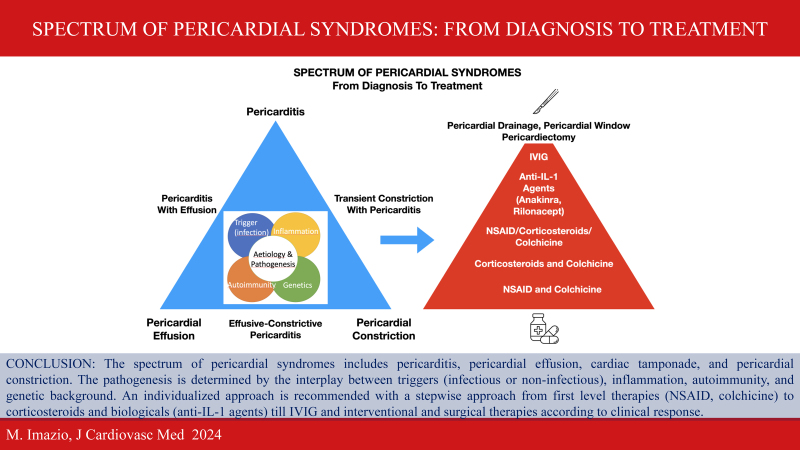

## What's new in the field of pericardial diseases

Pericardial diseases are not uncommon, but have been the Cinderella of Cardiovascular Medicine for decades, being at the boundaries of different medical specialties, and with limited published data to support evidence-based approaches to diagnosis and therapy. More recently, a growing interest has developed in the management of pericardial diseases. This is especially because of first dedicated guidelines,^1,2^ prospective cohort studies,^3–13^ and randomized controlled trials,^14–20^ which have introduced a new evidence-based approach to the diagnosis and therapy of these conditions and also new diagnostic tools, such as cardiac magnetic resonance (CMR) and multimodality imaging,^21,22^ as well as new therapeutic options^23–25^ (e.g. colchicine and more recently anti-IL-1 agents). The main novelties for the diagnosis and treatment of pericardial diseases are summarized in Table 1.

**Table 1:** Main novelties for the diagnosis and therapy of pericardial diseases from 2025 European Society of Cardiology guidelines on the management of pericardial diseases

Epidemiology	
Pathogenesis	New evidence on the interplay between genetic predisposition, autoinflammatory mechanisms, and autoimmunity for recurrent pericarditis^12,26–28^
Diagnosis	ECG changes (especially ST-segment elevation) as clues for concomitant myocarditis and not a simple diagnostic criterion for pericarditis^9^ The inflamed pericardium is thickened and neovascularized with contrast-enhancement on CT and CMR^21,29^ CMR is a diagnostic tool for the noninvasive diagnosis of pericarditis, for follow-up, helping to assess the best duration of anti-inflammatory therapies in a single patient^22,29^
Therapy	Anti-IL-1 agents as a new class of drugs for patients with colchicine resistance, corticosteroid dependence and evidence of systemic inflammation by elevation of C-reactive protein^4,18–20,24,25^ An inflammatory phenotype at presentation predicts a better response to specific treatments (colchicine and anti-IL-1 agents)^30^ Beta blockers can be considered on top of standard anti-inflammatory therapies to achieve a better control of symptoms in patients with pericarditis and heart rate >70 bpm^7^ Colchicine is well tolerated and useful for myopericarditis as for simple pericarditis^13^
Prognosis	Extension of pericardial LGE predicts the duration of the disease^22,27^ Incessant course of pericarditis is associated with a worse outcome and a greater risk of developing constrictive pericarditis in a few months^8^
Age and gender issues	Younger patients have more commonly an inflammatory presentation with more recurrences^31^ Updated indications for prepregnancy counselling, and drug treatments during pregnancy and lactation^32^

CMR, cardiac magnetic resonance; CT, computed tomography; LGE, late gadolinium enhancement.

## The spectrum of pericardial syndromes

**Fig. 1:**
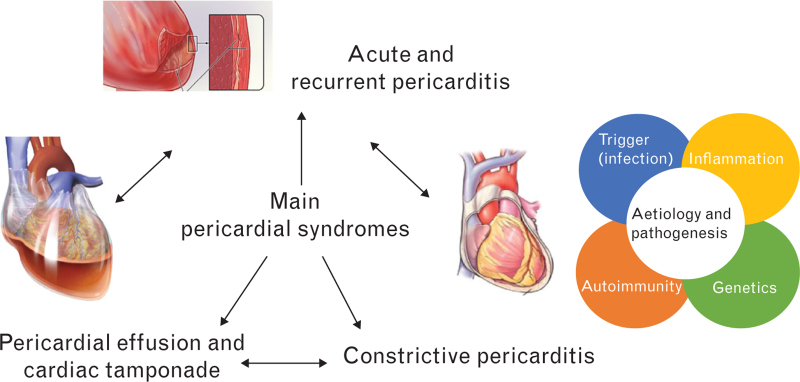
The spectrum of pericardial syndromes and the interplay between an infectious or noninfectious agent, genetic predisposition, inflammation, and autoimmunity in the pathogenesis of pericarditis.

The spectrum of pericardial syndromes includes pericarditis, pericardial effusion, cardiac tamponade, and constrictive pericarditis. Pericardial syndromes are manifestations of pericardial diseases that can be grouped into specific types of presentation, including signs and symptoms and having peculiar diagnostic, therapeutic, and prognostic features. Each syndrome can evolve into another one or have a complicated course (Fig. 1). In up to 10% of cases, pericarditis can show a transient constrictive physiology in the acute phase because of increased stiffness of the inflamed pericardium. Pericarditis may also progress to permanent constriction,^33,34^ or evolve into cardiac tamponade.^35,36^

All pericardial syndromes share the same mechanisms. Most cases are induced by an infectious agent, usually presumed to be viral in developed countries, but mainly tuberculosis in developing countries. Aetiological agents, either infectious or noninfectious, may cause inflammation of the pericardium (pericarditis) and increased production of pericardial fluid (inflammation), or in some cases reduced reabsorption (pericardial effusion). Constriction represents the final pathway of different pericardial syndromes (pericarditis, pericardial effusion with organization).^37^

The pathogenesis of pericardial diseases is more complex than previously suspected. This has been especially demonstrated for pericarditis.^26,38^ About 10% of patients with recurrent pericarditis have a positive family history of pericarditis, and 5–10% of cases have a defined genetic background especially represented by monogenic autoinflammatory conditions.^26,27,28,39^ Moreover, the striking response of patients with an inflammatory phenotype (fever and/or elevation of C-reactive protein with recurrences) to anti-IL-1 agents suggests the importance of individual inflammatory response and genetic background.^18–20,24–26^ In addition, many patients with recurrent pericarditis have either nonspecific autoantibodies, such as antinuclear antibodies or heart-specific autoantibodies, suggesting the importance of immune-mediated factors.^40,41^ On this basis, it seems evident that an infectious or noninfectious trigger may elicit a less or more pronounced inflammatory response according to the individual genetic, inflammatory, and immune profile. This notion may provide a theoretical framework for a more tailored treatment for these patients.^30,38^

## Acute and recurrent pericarditis

**Fig. 2:**
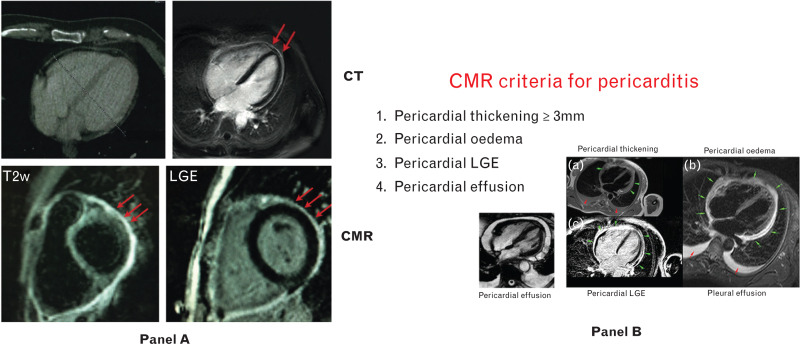
The inflamed pericardium is neovascularized and becomes contrast-enhanced on computed tomography and cardiac magnetic resonance (panel a). Proposed cardiac magnetic resonance (CMR) criteria for pericarditis (panel b).

Current diagnostic criteria for pericarditis are mainly clinical (Table 2).^2^ ECG changes, especially widespread ST-segment elevation, have been historically criteria to support the diagnosis of pericarditis. However, the pericardium is electrically silent, and evidence of ECG changes implies subepicardial involvement and should prompt a clinical evaluation to exclude concomitant myocarditis.^9^ Nowadays improvement in diagnostic capabilities allows the noninvasive evaluation of pericardial inflammation by means of CMR. The same sequences adopted for the evaluation of myocarditis can be used to assess pericardial oedema (T2-weighted images) and pericardial late gadolinium enhancement (LGE). The inflamed pericardium is neovascularized and becomes contrast-enhanced by using computed tomography (CT) and CMR (Fig. 2). On this basis, diagnostic criteria for pericarditis on CMR include: pericardial thickening, pericardial oedema on T2w-imaging, pericardial LGE, and pericardial effusion.^21^ Moreover, the extent of pericardial LGE has prognostic implications and may be considered to follow pericardial inflammation and establish the optimal duration of anti-inflammatory medical therapy.^10^

**Table 2:** Diagnostic criteria for pericarditis

Pericarditis	Current diagnostic criteria
Acute episode	Inflammatory pericardial syndrome to be diagnosed with at least two of the four following criteria: Pericarditic chest pain Pericardial rubs New widespread ST-elevation or PR depression on ECG Pericardial effusion (new or worsening) Additional supporting findings: Elevation of markers of inflammation (i.e. C-reactive protein, erythrocyte sedimentation rate, and white blood cell count) Evidence of pericardial inflammation by an imaging technique (computed tomography, cardiac magnetic resonance)

Incessant, persistent or new symptoms before clinical remission and withdrawal of medical therapy for pericarditis; recurrent, new acute episode after a clinical remission and withdrawal of previous anti-inflammatory therapy.

Medical therapy of pericarditis should be tailored and guided by the pathogenesis. Unfortunately, most cases remain idiopathic and empiric anti-inflammatory therapy is adopted to control symptoms and reduce the risk of recurrences.

Major advances in medical therapy of pericarditis include: emphasis on the importance of exercise restriction and heart rate control for clinical remission; use of combination therapies with NSAIDs, colchicine, and corticosteroids; implementation of anti-IL-1 agents for corticosteroid-dependent and colchicine-resistant cases; personalization of therapy based in presentation phenotype (inflammatory vs. non-inflammatory); and monitoring of disease activity by multimodality imaging, especially CMR to individualize the duration of therapy.

Exercise restriction is recommended in all cases until symptom resolution and clinical remission. Pharmacological control of the heart rate can be achieved in symptomatic patients with elevated heart rate (>70 bpm), despite anti-inflammatory therapies, by the use of beta blockers.^7^ First level treatments include NSAIDs and colchicine (Table 2). For those not responding to this first level therapy, low-dose corticosteroids (e.g. prednisone 0.2–0.5 mg/kg/day) should be considered in association with colchicine. Corticosteroids may have specific indication and could be first choices in specific settings: patients already on maintenance therapy with these drugs for a systemic inflammatory disease; postpericardiotomy syndromes; post-vaccine; concomitant use of anticoagulants; renal failure; pregnancy, especially after week 20.^42–45^ More difficult cases can be managed with a triple therapy combining a NSAID, low-dose corticosteroids, and colchicine. Combination therapy is common practice in different settings of cardiology (systemic hypertension, ischemic heart disease, heart failure, and hypercholesterolemia) and using low doses of combined drugs is usually more efficacious and safer than increasing the dosing of a single agent. A slow tapering of corticosteroids is recommended after clinical remission (e.g. reducing the daily dose of prednisone by 1.25–2.5 mg every 1–2 weeks).^45^ Patients with incessant pericarditis (continued symptoms without remission) can directly progress to constrictive pericarditis in a few months and warrant careful monitoring and aggressive treatment to prevent this unfavourable outcome.^8^

Patients not responding to NSAID, colchicine, and corticosteroids should be considered for anti-IL-1 agents that have been demonstrated to be particularly efficacious for corticosteroid-dependent, colchicine-resistant cases with elevation of C-reactive protein.^18–20,25^ Detailed reviews with specific notes for prescription of these agents in specific European countries are available.^24,46^ Anakinra is available on prescription within the National Healthcare system in Italy for patients with recurrent pericarditis, corticosteroid dependence, and colchicine resistance with elevation of C-reactive protein.^46^ Rilonacept has been registered in the United States with the Food and Drug Administration (FDA) indication based on the RHAPSODY trial.^19^

**Fig. 3:**
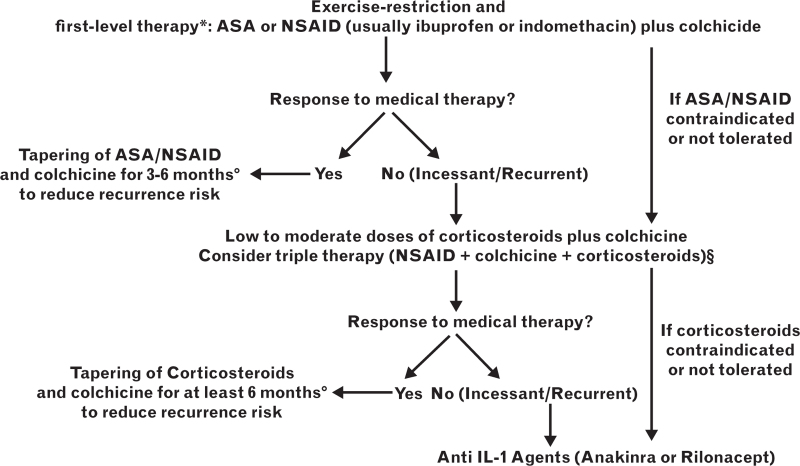
Proposed algorithm for medical therapy of pericarditis. Footnote ^∗^ASA is first option if patients already on antiplatelet therapy with ASA, ibuprofen usually preferred as first NSAID. Try more than one agent to evaluate response. ^°^Colchicine is recommended to prevent recurrences. Consider at least 3 months for first episode of pericarditis and at least 6 months for incessant/recurrent cases. Tapering is recommended to reduce the persistence/recurrence of symptoms. Tapering is slower for corticosteroids (e.g. decrease of the dose of prednisone by 1.25–2.5 mg every 1–2 weeks after clinical remission). ^§^Low to moderate dose of corticosteroids are prednisone 0.2–0.5 mg/kg/day or equivalent doses of alternative corticosteroid.

A stepwise algorithm is proposed in Fig. 3.

## Pericardial effusion and cardiac tamponade

Pericardial effusion can be defined as an abnormal accumulation of pericardial fluid that can be visualized by echocardiography (the first echocardiographic sign of very mild effusion is systodiastolic separation on M-mode recordings, whereas a simple systolic separation may be considered physiologic). A useful classification of pericardial effusion is the semiquantitative assessment of the largest telediastolic space in different echocardiographic views. The effusion is small with a size less than 10 mm, moderate between 10 and 20 mm, and large if greater than 20 mm.^47–49^

Pericardial effusion is a diagnostic criterion for pericarditis but is associated with pericarditis in a limited number of cases, often associated with elevation of inflammatory markers and other clinical criteria for pericarditis (Table 3).

**Table 3:** Dosing and duration of medical therapy for pericarditis

Therapy	Line	Dosing	Duration	Tapering	LOE
Aspirin°	First	750–500 mg three times daily	1–2 weeks	Weekly in 3–4 weeks	B
Ibuprofen°	First	600–800 mg three times daily	1–2 weeks	Weekly in 3–4 weeks	B
Indomethacin	First	25–50 mg three times daily	1–2 weeks	Weekly in 3–4 weeks	B
Colchicine°	First	0.5 once daily (<70 kg or severe renal impairment) or 0.5 mg twice daily	3–6 months	May be considered	A
Prednisone	Second	0.2–0.5 mg/kg/day	2–4 weeks	Several months	B
Anti-IL-1 agents: Anakinra Rilonacept	Third	1–2 mg/kg/day up to 100 mg/day in adults 320 mg/day once followed by 160 mg weekly	At least 3–6 months >12 months	Needed Probably not necessary	A
Azathioprine	Third/fourth	Starting with 1 mg/kg day then gradually increased to 2–3 mg/kg/day	Several months	Several months	C
IVIG	Third/fourth	400-500 mg/Kg iv daily for 5 days	5 days	Not required	C

A, based on systematic reviews or multiple RCTs; B, single RCT or observational studies; C, case reports and experts’ opinion; IVIG, intravenous immunoglobulin; LOE, level of evidence based on published evidence.

Recognizing pericardial effusion without pericarditis is crucial as the treatment for this condition is essentially the treatment of the cause (e.g. cancer, systemic inflammatory disease, metabolic condition), and empiric anti-infiammatory therapy is not effective in this setting.

An isolated pericardial effusion may be asymptomatic or have symptoms related to the underlying disease or related to the effusion itself, if moderate to large. Moreover, a pericardial effusion may be isolated, but it is often (50–60% of cases) associated with a systemic or underlying disease. The prognosis is essentially related to the cause of the effusion.^50^

The speed of its accumulation is critical for the time course of symptoms. As the pericardium is rather inelastic (as witnessed by a steep relationship between pressure and volume), a rapid accumulating pericardial effusion reaches the limit of pericardial stretch very soon with low volumes, whereas slowly accumulating fluid reaches the limit of pericardial stretch only for a huge volume as big as 1–2 l without the development of cardiac tamponade.^47–49^

The relative frequency of different causes depends on potential selection biases (geographic area, hospital setting, diagnostic testing). Nevertheless, the majority of cases remain ‘idiopathic’ (about 50% of cases) that is without a precise aetiological definition after the diagnostic workup. In clinical practice, the most common causes to be considered (in the absence of specific clues from the patient) include: pericarditis and infectious causes (15–30%), especially tuberculosis (main cause in developing countries with >60% of cases), cancer (10–25%), iatrogenic causes including postcardiac injury syndromes (15–20%), and connective tissue disease (5–15%).^51–54^

A triage has been proposed to evaluate the need for urgent or delayed pericardiocentesis in the setting of cardiac tamponade.^35,36,55^ This scoring system is based on three main classes of criteria: the aetiology, the clinical presentation, and imaging findings. A total score of 6 indicates the need for urgent pericardiocentesis.^56^Table 4 summarizes the proposed criteria for the evaluation of patients with cardiac tamponade.

**Table 4:** Criteria for the triage of cardiac tamponade according to probability of progression to cardiac tamponade

Aetiology	Clinical presentation	Imaging
Likely to progress		
Malignancy (2) Tuberculosis (2) Recent radiotherapy (1) Recent viral infection (1) Recurrent effusion (1) Previous pericardiocentesis (1) Chronic terminal renal failure (1) Immunodeficiency (1) Immunodepression (1) Iatrogenic haemopericardium (1) Posttraumatic aetiology (1) AMI mechanical complication (1) Type A aortic dissection (1)	Dyspnoea or tachypnea (1) Orthopnea, no rales (3) Hypotension (1) Distented jugular veins (1) Progressive tachycardia (1) Oliguria (1) Pulsus paradoxus (2) Rapid worsening symptoms (2)	Cardiomegaly (chest x-ray) (1) ECG electrical alternans (0.5) Microvoltages on ECG (0.5) Large PE >2 cm (3) Moderate PE (1–2 cm) (1) Right atrial collapse (1) IVC plethora (1.5) Right ventricular collapse (1.5) Left atrial collapse (2) Mitral/tricuspid respiratory flow variations (1) Swinging heart (1)
Rarely progress		
Post-AMI pericarditis (0) Chronic pericardial effusion (0) Hypothyroidism (−1) Systemic autoinflammatory dis (−1)	Low degree fever (0.5) Distant heart sounds (0.5) Pericardial chest pain (0.5) Normal blood pressure (−1) Slow evolution of disease (−1)	Normal chest x-ray (−1) Normal ECG (−1) Small pericardial effusion (−1) No trauma or recent intervention (−1)
Never progress		
Pericardial transudate (0) Heart failure (0) Pulmonary hypertension (0) Pregnancy (0)	Pulmonary oedema (0)	Trivial pericardial effusion, only systolic (0)

Each feature should be evaluated in the single patient. Numbers in parenthesis are points in scoring system for each feature, if present. If the total scoring is greater than 6, patients have indication for immediate pericardiocentesis. AMI, acute myocardial infarction; dis, disease; PE, pericardial effusion; IVC plethora: IVC size greater than 2.5 cm with less than 50% inspiratory collapse. Clinical case: a patient with dyspnoea (score 1), rapid worsening symptoms (score 2), microvoltages and electrical alternans on ECG (score 0.5 + 0.5), large pericardial effusion (score 3) with right atrial collapse and swinging heart on echocardiography (score 1 + 1), and cardiomegaly on chest x-ray (score 1) in the setting of a systemic inflammatory disease (score −1) has a total score of 1 + 2 + 0.5 + 0.5 + 3 + 1 + 1 − 1 = 9 with indication for immediate pericardiocentesis.

## Constriction and constrictive pericarditis

The final stage of any pathological process affecting the pericardium is represented by fibrosis with possible thickening (about 80% of cases) and calcification leading to a loss of pericardial elasticity and a constrictive effect of the pericardium on cardiac chambers, thus impairing diastolic filling in mid-late diastole, regardless of the different initial causes (e.g. infections, trauma, cardiac surgery, and radiation). This can occur with ongoing inflammation (constrictive pericarditis) or without residual inflammation (constriction).^37^ Constrictive evolution is rare in the setting of acute and recurrent pericarditis in the absence of specific aetiologies (e.g. tuberculosis and purulent forms) but has been reported for incessant pericarditis within a few months.^8^

A modern approach to the diagnosis of constriction considers multimodality imaging. The diagnosis of constriction combines clinical findings, mimicking right heart failure, and echocardiographic signs, and should be integrated with the evaluation of calcifications with their extension, by CT, and search for inflammation by CMR. Pericardial thickening can be evaluated by CT or CMR.^37,58^ Cardiac catheterization is no more essential for the diagnosis if the results of noninvasive imaging are straightforward, but should be considered for doubtful cases, when the diagnosis cannot be reached by means of multimodality imaging (echo, CT, and CMR).

It should be noted that constrictive physiology can be associated with pericardial inflammation (constrictive pericarditis) or not (constriction without pericarditis). The recognition of this difference has emerged to be of paramount importance for clinical management. Moreover about 10% of cases with acute pericarditis may show a constrictive physiology that is usually reversible (transient constrictive pericarditis) with medical therapy and caused by increased stiffness of the inflamed pericardium.^34^

Constrictive pericarditis is the correct terminology to address constrictive physiopathology associated with inflammation. This form should be treated for at least 3 months with anti-inflammatory therapy before considering cardiac surgery. In cases refractory to conventional therapies with NSAID, colchicine, and corticosteroids, the efficacy of anti-IL-1 agents has been demonstrated, and especially anakinra has been evaluated in this setting.^33^

If anti-inflammatory therapy fails or a chronic constriction is present, the final therapy is pericardiectomy, at least anterior pericardiectomy, but as complete as possible. Recent studies have demonstrated that the use of cardiopulmonary bypass (CPB) during pericardiectomy does not increase procedural risk or negatively impact survival. Radical resection of the pericardium, facilitated by CPB, helps minimize the risk of recurrent constrictions and the need for reinterventions.^57^ Early surgery can achieve better results that advanced cases with possible secondary involvement of the myocardium and extensive renal and hepatic impairment.

## Future perspectives

Pericardial disease management is now on the road of evidence-based medicine, and great improvements have been made in the noninvasive diagnosis of pericardial inflammation and medical therapy of corticosteroid-dependent and colchicine-resistant cases of recurrent pericarditis with an inflammatory phenotype. Nevertheless, the knowledge of the pathogenesis of complicated cases is only at the beginning, and additional research is needed to address the complex interplay between an infectious or noninfectious trigger, genetic predisposition, inflammation, and autoimmunity. Moreover, additional drugs are needed to better treat refractory cases to NSAID, colchicine, and corticosteroids.

A new emerging issue is the problem of anti-IL-1 agent dependence after the starting of these drugs in patients with corticosteroid-dependent, colchicine-resistant recurrent pericarditis. Although these patients can reach a stable remission on therapy and usually are able to withdraw corticosteroids, they may become dependent on continued therapy with anti-IL-1 agents to maintain stable remission. At present, the reason for this phenomenon is unknown and it is currently under investigation.

Teams of cardiologists, cardiac surgeons, rheumatologists, internal medicine physicians, and radiologists are becoming increasingly important in order to provide the best diagnostic and therapeutic approach for patients with pericardial diseases, highlighting the importance of a multidisciplinary team also for pericardial diseases.

Another issue is represented by the significance of persistent pericardial inflammation by CMR. Pericardial LGE is common after cardiac surgery and has been reported in about 40% of cases, even in asymptomatic cases.^59^

The clinical significance of this finding in the long term is unknown (including the risk of developing constriction), especially in patients with recurrent pericarditis.^60^

Ongoing research will be focused on a better understanding of the pathogenesis of pericardial diseases (genetic predisposition, autoinflammatory, and autoimmunity mechanisms), and development of new therapeutic tools for the treatment of refractory cases to conventional therapies providing more individualized approaches targeted at the single patient.

## Conclusion

In recent years, major advances have been achieved in multimodality imaging of pericardial diseases, risk stratification of cases for hospital admission, and treatment of corticosteroid-dependent cases with a new class of drugs, the anti-IL-1 agents. Ongoing research is needed to better understand the complex pathogenesis of complicated cases with the interplay between genetic predisposition, inflammation, autoimmunity, and infectious or noninfectious triggers in order to develop more successful medical therapies for patients.

### Conflicts of interest

There are no conflicts of interest.

## References

[1] Maisch B Seferović PM Ristić AD. Task Force on the Diagnosis and Management of Pericardial Diseases of the European Society of Cardiology. Guidelines on the diagnosis and management of pericardial diseases executive summary; the Task force on the diagnosis and management of pericardial diseases of the European society of cardiology. *Eur Heart J* 2004; 25:587–610. 15120056 10.1016/j.ehj.2004.02.002

[2] Adler Y Charron P Imazio M. ESC Scientific Document Group. 2015 ESC Guidelines for the diagnosis and management of pericardial diseases: the Task Force for the Diagnosis and Management of Pericardial Diseases of the European Society of Cardiology (ESC)Endorsed by: the European Association for Cardio-Thoracic Surgery (EACTS). *Eur Heart J* 2015; 36:2921–2964. 26320112 10.1093/eurheartj/ehv318 PMC7539677

[3] Imazio M Demichelis B Parrini I. Day-hospital treatment of acute pericarditis: a management program for outpatient therapy. *J Am Coll Cardiol* 2004; 43:1042–1046. 15028364 10.1016/j.jacc.2003.09.055

[4] Imazio M Andreis A De Ferrari GM. Anakinra for corticosteroid-dependent and colchicine-resistant pericarditis: the IRAP (International Registry of Anakinra for Pericarditis) study. *Eur J Prev Cardiol* 2020; 27:956–964. 31610707 10.1177/2047487319879534

[5] Lazarou E Lazaros G Antonopoulos AS. A risk score for pericarditis recurrence. *Eur J Clin Invest* 2021; 51:e13602. 34050527 10.1111/eci.13602

[6] Imazio M Andreis A Lubian M. The Torino Pericarditis Score: a new-risk stratification tool to predict complicated pericarditis. *Intern Emerg Med* 2021; 16:1921–1926. 34275095 10.1007/s11739-021-02803-y

[7] Imazio M Andreis A Agosti A. Usefulness of beta-blockers to control symptoms in patients with pericarditis. *Am J Cardiol* 2021; 146:115–119. 33539856 10.1016/j.amjcard.2021.01.032

[8] Imazio M Pivetta E Andreis A. Incessant pericarditis as a risk factor for complicated pericarditis and hospital admission. *Circulation* 2021; 143:401–402. 33493032 10.1161/CIRCULATIONAHA.120.051156

[9] Imazio M Squarotti GB Andreis A. Diagnostic and prognostic role of the electrocardiogram in patients with pericarditis. *Heart* 2022; 108:1474–1478. 35523541 10.1136/heartjnl-2021-320443

[10] Kumar AK Yesilyaprak A Furqan MM. Prognostic value of inflammatory markers in idiopathic recurrent pericarditis. *J Am Coll Cardiol* 2022; 79:1644–1645. 35450584 10.1016/j.jacc.2022.02.016

[11] Conte E Leoni O Ammirati E Imazio M Brucato A. Incidence of myocarditis and pericarditis considered as separate clinical events over the years and post-SARS-CoV2 vaccination in adults and children. *Eur J Intern Med* 2023; 115:140–142. 37311686 10.1016/j.ejim.2023.06.002 PMC10250150

[12] Pisacreta AM Mascolo R Nivuori M. Acute pericarditis with pleuropulmonary involvement, fever and elevated C-reactive protein: a systemic autoinflammatory disease? A cohort study. *Eur J Intern Med* 2023; 113:45–48. 37069014 10.1016/j.ejim.2023.03.034

[13] Collini V De Martino M Andreis A. Efficacy and safety of colchicine for the treatment of myopericarditis. *Heart* 2024; 110:735–739. 38238076 10.1136/heartjnl-2023-323484 PMC11103299

[14] Imazio M Bobbio M Cecchi E. Colchicine in addition to conventional therapy for acute pericarditis: results of the COlchicine for acute PEricarditis (COPE) trial. *Circulation* 2005; 112:2012–2016. 16186437 10.1161/CIRCULATIONAHA.105.542738

[15] Imazio M Bobbio M Cecchi E. Colchicine as first-choice therapy for recurrent pericarditis: results of the CORE (COlchicine for REcurrent pericarditis) trial. *Arch Intern Med* 2005; 165:1987–1991. 16186468 10.1001/archinte.165.17.1987

[16] Imazio M Brucato A Cemin R. ICAP Investigators. A randomized trial of colchicine for acute pericarditis. *N Engl J Med* 2013; 369:1522–1528. 23992557 10.1056/NEJMoa1208536

[17] Imazio M Belli R Brucato A. Efficacy and safety of colchicine for treatment of multiple recurrences of pericarditis (CORP-2): a multicentre, double-blind, placebo-controlled, randomised trial. *Lancet* 2014; 383:2232–2237. 24694983 10.1016/S0140-6736(13)62709-9

[18] Brucato A Imazio M Gattorno M. Effect of anakinra on recurrent pericarditis among patients with colchicine resistance and corticosteroid dependence: the AIRTRIP Randomized Clinical Trial. *JAMA* 2016; 316:1906–1912. 27825009 10.1001/jama.2016.15826

[19] Klein AL Imazio M Cremer P. RHAPSODY Investigators. Phase 3 trial of interleukin-1 trap rilonacept in recurrent pericarditis. *N Engl J Med* 2021; 384:31–41. 33200890 10.1056/NEJMoa2027892

[20] Myachikova VY Maslyanskiy AL Moiseeva OM. Treatment of idiopathic recurrent pericarditis with goflikicept: phase II/III study results. *J Am Coll Cardiol* 2023; 82:30–40. 37380301 10.1016/j.jacc.2023.04.046

[21] Imazio M Pivetta E Palacio Restrepo S. Usefulness of cardiac magnetic resonance for recurrent pericarditis. *Am J Cardiol* 2020; 125:146–151. 31711636 10.1016/j.amjcard.2019.09.026

[22] Jain V Chhabra G Chetrit M. Role of noninvasive multimodality imaging in autoimmune pericarditis. *Eur Heart J Cardiovasc Imaging* 2021; 22:1228–1240. 34333596 10.1093/ehjci/jeab131

[23] Imazio M Nidorf M. Colchicine and the heart. *Eur Heart J* 2021; 42:2745–2760. 33961006 10.1093/eurheartj/ehab221 PMC8294843

[24] Imazio M Lazaros G Gattorno M. Antiinterleukin-1 agents for pericarditis: a primer for cardiologists. *Eur Heart J* 2022; 43:2946–2957. 34528670 10.1093/eurheartj/ehab452 PMC9375710

[25] Imazio M Andreis A Piroli F. Antiinterleukin 1 agents for the treatment of recurrent pericarditis: a systematic review and meta-analysis. *Heart* 2021; 107:1240–1245. 33737453 10.1136/heartjnl-2020-318869

[26] Imazio M Faletra F Zucco J. Genetic variants in patients with recurrent pericarditis. *J Cardiovasc Med* 2024; 25:799–804. 10.2459/JCM.0000000000001669 PMC11581433 39347728

[27] Peet CJ Rowczenio D Omoyinmi E. Pericarditis and autoinflammation: a clinical and genetic analysis of patients with idiopathic recurrent pericarditis and monogenic autoinflammatory diseases at a national referral center. *J Am Heart Assoc* 2022; 11:e024931. 35658515 10.1161/JAHA.121.024931 PMC9238712

[28] Thorolfsdottir RB Jonsdottir AB Sveinbjornsson G. Danish Blood Donor Study Genomic Consortium. Variants at the interleukin 1 gene locus and pericarditis. *JAMA Cardiol* 2024; 9:165–172. 38150231 10.1001/jamacardio.2023.4820 PMC10753444

[29] Kumar S Khubber S Reyaldeen R. Advances in imaging and targeted therapies for recurrent pericarditis: a review. *JAMA Cardiol* 2022; 7:975–985. 35976625 10.1001/jamacardio.2022.2584

[30] Imazio M Mardigyan V Andreis A Franchin L De Biasio M Collini V. New developments in the management of recurrent pericarditis. *Can J Cardiol* 2023; 39:1103–1110. 37075863 10.1016/j.cjca.2023.04.008

[31] Lazaros G Antonopoulos AS Lazarou E. Age- and sex-based differences in patients with acute pericarditis. *Eur J Clin Invest* 2021; 51:e13392. 32857868 10.1111/eci.13392

[32] Serati L Mardigyan V Dominioni CC. Pericardial diseases in pregnancy. *Can J Cardiol* 2023; 39:1067–1077. 37086835 10.1016/j.cjca.2023.04.010

[33] Andreis A Imazio M Giustetto C Brucato A Adler Y De Ferrari GM. Anakinra for constrictive pericarditis associated with incessant or recurrent pericarditis. *Heart* 2020; 106:1561–1565. 32868281 10.1136/heartjnl-2020-316898

[34] Baritussio A Giordani AS Iliceto S Marcolongo R Caforio ALP. Transient pericardial constriction: a not so rare entity. *Int J Cardiol* 2023; 390:131225. 37524124 10.1016/j.ijcard.2023.131225

[35] Imazio M De Ferrari GM. Cardiac tamponade: an educational review. *Eur Heart J Acute Cardiovasc Care* 2020; 10:102–109. 32628038 10.1177/2048872620939341

[36] Adler Y Ristić AD Imazio M. Cardiac tamponade. *Nat Rev Dis Primers* 2023; 9:36. 37474539 10.1038/s41572-023-00446-1

[37] Gillombardo CB Hoit BD. Constrictive pericarditis in the new millennium. *J Cardiol* 2024; 83:219–227. 37714264 10.1016/j.jjcc.2023.09.003

[38] Bonaventura A Vecchié A Mauro AG Brucato AL Imazio M Abbate A. An update on the pathophysiology of acute and recurrent pericarditis. *Panminerva Med* 2021; 63:249–260. 33337127 10.23736/S0031-0808.20.04205-6

[39] Cantarini L Lucherini OM Brucato A. Clues to detect tumor necrosis factor receptor-associated periodic syndrome (TRAPS) among patients with idiopathic recurrent acute pericarditis: results of a multicentre study. *Clin Res Cardiol* 2012; 101:525–531. 22311714 10.1007/s00392-012-0422-8

[40] Imazio M Brucato A Doria A. Antinuclear antibodies in recurrent idiopathic pericarditis: prevalence and clinical significance. *Int J Cardiol* 2009; 136:289–293. 18674829 10.1016/j.ijcard.2008.05.020

[41] Caforio AL Brucato A Doria A. Antiheart and antiintercalated disk autoantibodies: evidence for autoimmunity in idiopathic recurrent acute pericarditis. *Heart* 2010; 96:779–784. 20448129 10.1136/hrt.2009.187138

[42] Avondo S Andreis A Casula M Biondi-Zoccai G Imazio M. Pharmacologic treatment of acute and recurrent pericarditis: a systematic review and meta-analysis of controlled clinical trials. *Panminerva Med* 2021; 63:314–323. 34738773 10.23736/S0031-0808.21.04263-4

[43] Imazio M Lazaros G. Corticosteroids for pericarditis: a warning but don’t throw the baby out with the bathwater. *Hellenic J Cardiol* 2019; 60:364–365. 30980882 10.1016/j.hjc.2019.04.002

[44] Imazio M Adler Y. Treatment with aspirin, NSAID, corticosteroids, and colchicine in acute and recurrent pericarditis. *Heart Fail Rev* 2013; 18:355–360. 22661042 10.1007/s10741-012-9328-9

[45] Imazio M Brucato A Cumetti D. Corticosteroids for recurrent pericarditis: high versus low doses: a nonrandomized observation. *Circulation* 2008; 118:667–671. 18645054 10.1161/CIRCULATIONAHA.107.761064

[46] Imazio M Lazaros G Gattorno M Abbate A Brucato A. Gli antagonisti dell’interleuchina-1: una nuova classe di farmaci per il trattamento della pericardite ricorrente. Una guida pratica per il cardiologo clinico [Antiinterleukin-1 agents: a new class of drugs for recurrent pericarditis: a practical guide for cardiologists]. *G Ital Cardiol (Rome)* 2021; 22:833–843. 34570117 10.1714/3666.36514

[47] Imazio M Adler Y. Management of pericardial effusion. *Eur Heart J* 2013; 34:1186–1197. 23125278 10.1093/eurheartj/ehs372

[48] Imazio M Gaido L Battaglia A Gaita F. Contemporary management of pericardial effusion: practical aspects for clinical practice. *Postgrad Med* 2017; 129:178–186. 28135875 10.1080/00325481.2017.1285676

[49] Lazaros G Imazio M Tsioufis P Lazarou E Vlachopoulos C Tsioufis C. Chronic pericardial effusion: causes and management. *Can J Cardiol* 2023; 39:1121–1131. 36773704 10.1016/j.cjca.2023.02.003

[50] De Filippo O Gatti P Rettegno S. Is pericardial effusion a negative prognostic marker? Meta-analysis of outcomes of pericardial effusion. *J Cardiovasc Med (Hagerstown)* 2019; 20:39–45. 30480582 10.2459/JCM.0000000000000720

[51] Imazio M Colopi M De Ferrari GM. Pericardial diseases in patients with cancer: contemporary prevalence, management and outcomes. *Heart* 2020; 106:569–574. 31980441 10.1136/heartjnl-2019-315852

[52] Avondo S Andreis A Casula M Imazio M. Update on diagnosis and management of neoplastic pericardial disease. *Expert Rev Cardiovasc Ther* 2020; 18:615–623. 32797759 10.1080/14779072.2020.1811087

[53] Chahine J Shekhar S Mahalwar G Imazio M Collier P Klein A. Pericardial involvement in cancer. *Am J Cardiol* 2021; 145:151–159. 33460602 10.1016/j.amjcard.2020.12.092

[54] Maranta F Cianfanelli L Grippo R Alfieri O Cianflone D Imazio M. Postpericardiotomy syndrome: insights into neglected postoperative issues. *Eur J Cardiothorac Surg* 2022; 61:505–514. 34672331 10.1093/ejcts/ezab449

[55] Ristić AD Imazio M Adler Y. Triage strategy for urgent management of cardiac tamponade: a position statement of the European Society of Cardiology Working Group on Myocardial and Pericardial Diseases. *Eur Heart J* 2014; 35:2279–2284. 25002749 10.1093/eurheartj/ehu217

[56] Maggiolini S De Carlini CC Imazio M. Evolution of the pericardiocentesis technique. *J Cardiovasc Med (Hagerstown)* 2018; 19:267–273. 29553993 10.2459/JCM.0000000000000649

[57] Moros D Zaki A Tong MZ. Surgical approaches for pericardial diseases: what is new? *Curr Cardiol Rep* 2023; 25:1705–1713. 37938424 10.1007/s11886-023-01986-4

[58] Lloyd JW Anavekar NS Oh JK Miranda WR. Multimodality imaging in differentiating constrictive pericarditis from restrictive cardiomyopathy: a comprehensive overview for clinicians and imagers. *J Am Soc Echocardiogr* 2023; 36:1254–1265. 37619909 10.1016/j.echo.2023.08.016

[59] Pavon AG Martinez Fernandez R Arangalage D. Prevalence of pericardial late gadolinium enhancement in patients after cardiac surgery: clinical and histological correlations. *Circ Cardiovasc Imaging* 2023; 16:e015606. 37988447 10.1161/CIRCIMAGING.123.015606

[60] Antonopoulos AS Vrettos A Androulakis E. Cardiac magnetic resonance imaging of pericardial diseases: a comprehensive guide. *Eur Heart J Cardiovasc Imaging* 2023; 24:983–998. 37207354 10.1093/ehjci/jead092

